# Regulation of the iron-deficiency response by IMA/FEP peptide

**DOI:** 10.3389/fpls.2023.1107405

**Published:** 2023-04-25

**Authors:** Ryo Tabata

**Affiliations:** Graduate School of Bioagricultural Sciences, Nagoya University, Nagoya, Japan

**Keywords:** iron deficiency response, IRON MAN/FE-UPTAKE-INDUCING PEPTIDE, Fe uptake, organ-to-organ communication, systemic Fe signaling

## Abstract

Iron (Fe) is an essential micronutrient for plant growth and development, participating in many significant biological processes including photosynthesis, respiration, and nitrogen fixation. Although abundant in the earth’s crust, most Fe is oxidized and difficult for plants to absorb under aerobic and alkaline pH conditions. Plants, therefore, have evolved complex means to optimize their Fe-acquisition efficiency. In the past two decades, regulatory networks of transcription factors and ubiquitin ligases have proven to be essential for plant Fe uptake and translocation. Recent studies in *Arabidopsis thaliana* (Arabidopsis) suggest that in addition to the transcriptional network, IRON MAN/FE-UPTAKE-INDUCING PEPTIDE (IMA/FEP) peptide interacts with a ubiquitin ligase, BRUTUS (BTS)/BTS-LIKE (BTSL). Under Fe-deficient conditions, IMA/FEP peptides compete with IVc subgroup bHLH transcription factors (TFs) to interact with BTS/BTSL. The resulting complex inhibits the degradation of these TFs by BTS/BTSL, which is important for maintaining the Fe-deficiency response in roots. Furthermore, IMA/FEP peptides control systemic Fe signaling. By organ-to-organ communication in Arabidopsis, Fe deficiency in one part of a root drives the upregulation of a high-affinity Fe-uptake system in other root regions surrounded by sufficient levels of Fe. IMA/FEP peptides regulate this compensatory response through Fe-deficiency-triggered organ-to-organ communication. This mini-review summarizes recent advances in understanding how IMA/FEP peptides function in the intracellular signaling of the Fe-deficiency response and systemic Fe signaling to regulate Fe acquisition.

## Introduction

Iron (Fe) is an essential element for all living organisms. Fe participates in many significant biological processes, including photosynthesis, electron transfer in respiration, and numerous enzymatic reactions (Marschner, 1995). Fe is the fourth most abundant element in the earth’s crust, but under aerobic and alkaline pH conditions, most Fe is oxidized to the insoluble form, ferric iron (Fe^3+^), and cannot be easily absorbed by plants. Therefore, plants have evolved two distinct iron acquisition strategies to adapt to the distribution of Fe in the soil (Römheld and Marschner, 1986). To acquire Fe, dicots and non-grass monocotyledonous plants use a reduction-based strategy called Strategy I, whereas grasses mainly use a chelation strategy named Strategy II (Römheld and Marschner, 1986).

In Strategy I, plants acidify the rhizosphere by releasing protons to promote Fe dissolution and mobilize Fe^3+^ by secreting small metabolites such as coumarins and flavins (Santi and Schmidt, 2009; Fourcroy et al., 2016; Robe et al., 2021). Ferric-chelate is reduced to ferrous iron (Fe^2+^) at the root cell membrane and absorbed into the epidermal cells *via* high-affinity Fe^2+^ transporters. In Arabidopsis, proton release to the rhizosphere is mediated by H^+^-ATPase 2 (AHA2) (Santi and Schmidt, 2009). Fe reduction and uptake are mediated by FERRIC REDUCTASE OXIDASE 2 (FRO2) and IRON-REGULATED TRANSPORTER 1 (IRT1), respectively (Eide et al., 1996; Robinson et al., 1999). In root cell membranes, AHA2, FRO2, and IRT1 are thought to form a complex to achieve efficient Fe uptake (Martín-Barranco et al., 2020). Secretion of coumarins is regulated by PLEIOTROPIC DRUG RESISTANCE 9 (PDR9)/ATP-BINDING CASSETTE G37 (ABCG37) transporters (Fourcroy et al., 2016), but the secretory transporters of flavins have not been identified. In contrast, in Strategy II of grasses, mugineic acids (MAs) are synthesized in the roots and are exported to the rhizosphere by the secretory TRANSPORTER OF MUGINEIC ACID 1 (TOM1) that chelates and solubilizes Fe^3+^ (Kobayashi et al., 2014). The resulting Fe^3+^-MA complex is absorbed by the YELLOW STRIPE 1 (YS1)/YELLOW STRIPE 1-like (YSL) transporter (Curie et al., 2009). Recent studies have revealed that some plants do not rely on only one strategy and can use both reduction and chelation strategies to acquire Fe. For example, rice plants can absorb Fe in the soil not only by Strategy II but also by the reduction-based strategy *via* OsIRT1 and OsIRT2 Fe^2+^ transporters (Ishimaru et al., 2006). In addition to MA, caffeic acid and protocatechuic acid are secreted into the rhizosphere of rice plants and hypothesized to be involved in the solubilization and reduction of Fe^3+^ to Fe^2+^ (Bashir et al., 2011).

**Figure 1:**
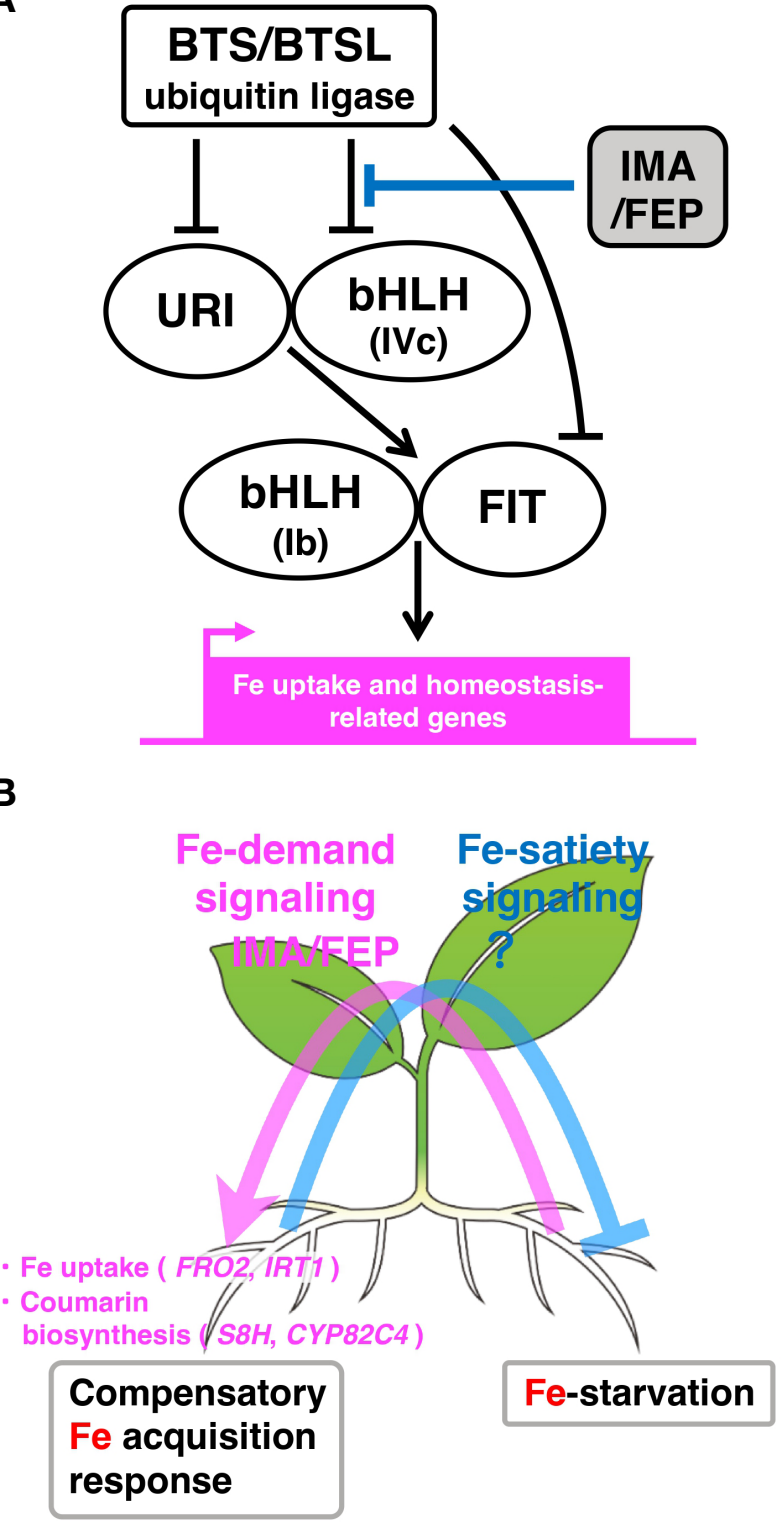
Model for IMA/FEP-mediated local Fe-deficiency response and systemic Fe signaling under heterogeneous Fe conditions. **(A)** IMA/FEP-mediated intracellular signaling of the local Fe-deficiency response. BTS/BTSL ubiquitin E3 ligases, which are potential Fe sensor proteins, negatively regulate local Fe-deficiency responses by degrading of IVc subgroup bHLH TFs, URI and FIT. IMA/FEP peptides compete with IVc subgroup bHLH TFs to interact with BTS/BTSL, which inhibits the degradation of these TFs by BTS/BTSL. The IVc subgroup bHLH TFs form heterodimers with URI to activate the bHLH TF-mediated signaling cascade for the upregulation of Fe uptake and homeostasis-related genes in response to Fe-deficiency. **(B)** Systemic Fe signaling under heterogeneous Fe conditions. Detection of Fe deficiency status in one region of the root (right side of the root) is transduced to shoots by an as-yet unknown mechanism, resulting in the upregulation of IMA/FEP peptide expression as a descending signal to roots (pink arrow: Fe-demand signaling). These putative shoot-derived peptides upregulate the expression of Fe uptake and coumarin biosynthesis genes in other root regions (left side of the root) to remediate the local Fe deficiency. Conversely, the putative Fe-satiety signal from the Fe-sufficient side is conveyed to the Fe-deficient side to suppress Fe uptake mechanisms (light blue line: Fe-satiety signaling).

Fe deficiency induces the expression of these key enzymes and transporter genes involved in Fe uptake at the transcriptional level (Kobayashi, 2019). The expression of these Fe-deficiency responsive genes in roots is regulated by a transcriptional regulatory network of transcription factors (TFs). In Arabidopsis, the master regulator, *FER-LIKE IRON DEFICIENCY-INDUCED TRANSCRIPTION FACTOR* (*FIT*), is a basic helix-loop-helix (bHLH) TF that regulates the expression of *AHA2*, *FRO2* and *IRT1* (Colangelo and Guerinot, 2004; Jakoby et al., 2004). The FIT protein interacts with other Ib subgroup bHLH TFs, specifically bHLH38, bHLH39, bHLH100 and bHLH101 (Wang et al., 2007; Yuan et al., 2008). The expression of *FIT* and *bHLH38*/*39*/*100*/*101* is induced by IVb subgroup bHLH TFs (bHLH121/URI) and IVc subgroup bHLH TFs (bHLH34, bHLH104, bHLH105/ILR3 and bHLH115) (Zhang et al., 2015; Li et al., 2016; Liang et al., 2017). In addition to regulation by TFs, the BRUTUS (BTS) and BTS-LIKE (BTSL) proteins, which are ubiquitin E3 ligases, interact with IVc subgroup bHLH TFs and FIT, targeting them for proteasomal degradation (Hindt et al., 2017; Kim et al., 2019; Liang et al., 2017; Rodríguez-Celma et al., 2019). Thus, BTS/BTSL negatively regulates Fe-deficiency responses by degrading of IVc subgroup bHLH TFs and FIT (**Figure 1A**).

The transcriptional network of ubiquitin ligases and a group of transcription factors has been detailed in the Fe-deficiency response in plants. With the discovery of the IRON MAN/FE-UPTAKE-INDUCING PEPTIDE (IMA/FEP) peptide in 2018, our understanding of the regulatory mechanisms of the Fe-deficiency response has taken a new turn (Grillet et al., 2018; Hirayama et al., 2018). This mini-review presents recent findings on the intracellular signaling of the Fe-deficiency response and Fe systemic signaling regulated by IMA/FEP peptides.

## IMA/FEP peptide functions in intracellular signaling in response to iron-starvation

IMA/FEP is an Fe deficiency-inducing peptide that is widely conserved in angiosperms. The IMA/FEP peptide contains about 50 amino acids and has a conserved sequence containing an aspartic acid-rich consensus motif in the C-terminal region. Eight IMA/FEP peptides are present in the Arabidopsis genome and two in rice. Overexpression of Arabidopsis *IMA1*/*FEP3* in tomato enhanced accumulation of Fe in fruits, supporting that the IMA/FEP-mediated mechanism is conserved among angiosperms. The expression of all Arabidopsis *IMA*/*FEP* genes is induced in response to Fe-starvation (Grillet et al., 2018; Tabata et al., 2022) and all individual *IMA*/*FEP* genes, except *IMA5* and *IMA8*, induce Fe uptake (Li et al., 2021). Among the Arabidopsis *IMA*/*FEP* genes, the expression of *IMA1*/*FEP3* and *IMA2*/*FEP2* is directly controlled by bHLH121/URI with bHLH105/ILR3 and its closest homologs (bHLH34, bHLH104 and bHLH115) (Gao et al., 2020). Recently, Arabidopsis IMA/FEP was reported to interact first with BTS ubiquitin ligase, followed by ubiquitination of IMA/FEP peptides and degradation by BTS to maintain Fe homeostasis (Li et al., 2021). Under Fe-deficient conditions, IMA/FEP peptides compete with IVc subgroup bHLH TFs to interact with BTS/BTSL, thereby inhibiting the degradation of these TFs by BTS/BTSL. Therefore, this competition is essential for maintaining the Fe-deficiency response in Arabidopsis roots (**Figure 1A**) (Li et al., 2021; Lichtblau et al., 2022; Vélez-Bermúdez and Schmidt, 2022). Despite recent progress in this area, the original event that activates this signaling cascade in Fe-deficiency response has not yet been identified and requires further examination.

In rice, as in Arabidopsis, the expression of *OsIMA1* and *OsIMA2* is also induced under Fe-deficient conditions; *OsIMA1* and *OsIMA2* overexpression in rice provided tolerance to Fe deficiency, and Fe accumulation occurred in leaves (Kobayashi et al., 2021). *OsIMA1* and *OsIMA2* expression is regulated by IVc subgroup bHLH TFs, such as OsbHLH058 and OsbHLH059. OsIMA/FEP peptides are degraded upon interaction with Haemerythrin motif-containing Really Interesting New Gene (RING)- and Zinc-finger protein (HRZ) proteins that are BTS ubiquitin ligase homologs (Kobayashi et al., 2021; Peng et al., 2022).

From the results of expression analyses using *IMA*/*FEP* inducible expression and overexpressing Arabidopsis plants, induction of *IMA*/*FEP* can activate the expression of Ib subgroup bHLH TFs (*bHLH38*, *bHLH39*, *bHLH100* and *bHLH101*), *FRO2* and *IRT1* genes for Fe reduction and uptake, the coumarin biosynthesis gene *SCOPOLETIN 8*-*HYDROXYLASE* (*S8H*), and *CYP82C4* for Fe chelation (Grillet et al., 2018; Hirayama et al., 2018; Gautam et al., 2021; Okada et al., 2022). Genes encoding proteins for Fe storage (*FERRITIN*) and vacuolar Fe transporters (*VACUOLAR IRON TRANSPORTER-LIKE*) are also upregulated in *IMA1*/*FEP3*-overexpressing plants (Grillet et al., 2018). Moreover, *OsIMA1*- or *OsIMA2*-overexpressing rice has enhanced expression of most of the known Fe-deficiency-inducible genes that are involved in Fe uptake and translocation, *i*.*e*., the Ib subgroup bHLH TFs, Fe^2+^ uptake genes, Fe^3+^-MA biosynthesis/uptake/translocation genes, and Fe-translocation-related genes (Kobayashi et al., 2021). Transcriptome data show that overexpression of one *OsIMA* gene induces the expression of another *OsIMA* gene, revealing a positive feedback loop for *IMA*/*FEP* expression in Fe-sufficient roots (Kobayashi et al., 2021). However, in Arabidopsis, the expression levels of other *IMA*/*FEP* genes were either not upregulated or downregulated in the overexpressors of *IMA1* (Grillet et al., 2018). These results indicate that IMA/FEP peptides induce several genes responsible for Fe uptake and translocation by roots to acquire adequate amounts of Fe in roots.

## The role of IMA/FEP peptide in systemic Fe signaling in response to iron-starvation

In addition to the plant response to local Fe-deficient conditions described so far, Fe absorption by plants is also regulated through organ-to-organ communication in response to a heterogeneous Fe environment in the soil. When sensing Fe deficiency in some roots, plants actively increase Fe absorption in other roots where sufficient Fe is present, maintaining a level of Fe acquisition sufficient for the entire plant (Vert et al., 2003; Shanmugam et al., 2015). Several studies have used split-root culture methods that mimic a heterogeneous nutrient distribution in the soil to analyze systemic Fe signaling in response to a heterogeneous Fe environment (Vert et al., 2003; Shanmugam et al., 2015; Tabata et al., 2022). These experiments showed that *FRO2* and *IRT1* were upregulated in roots on the +Fe side of split-root culture relative to control conditions, and downregulated in −Fe side roots relative to homogeneous Fe-deficient conditions (Vert et al., 2003; Shanmugam et al., 2015; Tabata et al., 2022), indicating that the Fe-demand signal from the −Fe side of split-root culture was conveyed to the +Fe side to upregulate the expression of *FRO2* and *IRT1*, and the Fe-satiety signal from the +Fe side of split-root culture was conveyed to the −Fe side to suppress the expression of *FRO2* and *IRT1*. Thus, two-way communication by Fe-demand and -satiety signals may be associated with systemic Fe signaling under heterogeneous Fe conditions (**Figure 1B**). Split-root analysis using Arabidopsis also revealed that *S8H* and *CYP82C4*, a group of coumarin biosynthetic enzymes that contribute to enhanced Fe absorption, are complementarily regulated through organ-to-organ communication (Tabata et al., 2022). Similar to the response to a heterogeneous Fe environment, nitrogen (N) absorption genes are complementarily upregulated in heterogeneous N environments (Ruffel et al., 2011). Some peptide factors that regulate the N responses have also been identified (Tabata et al., 2014; Ohkubo et al., 2017). Following N deprivation in some roots, C-terminally encoded peptides (CEPs) are translocated to the shoots through the xylem, where they are recognized by two CEP receptors (Tabata et al., 2014). Within the leaf vascular tissue, this interaction leads to the upregulation of CEP Downstream (CEPD) peptides (glutaredoxin-like polypeptides) that translocate toward the roots to control expression of the ${\text{NO}}_{3}^{-}$ transporter *NRT2.1* (Okamoto et al., 2016; Ohkubo et al., 2017). Analogous to the response for a heterogeneous N environment, IMA/FEP is considered to be a potential descending shoot-to-root signal for organ-to-organ communication between shoots and roots in the heterogeneous Fe environments (Grillet et al., 2018; Tabata et al., 2022). Arabidopsis *IMA*/*FEP* expression is strongly induced in leaves when plants sense root Fe deficiency (Grillet et al., 2018; Tabata et al., 2022). Grafting experiments have also shown that when *IMA*/*FEP* expression is successfully induced in leaves, gene expression levels for *IRT1* and *S8H*, and ferric chelate reductase (FCR) in roots are restored (Grillet et al., 2018; Tabata et al., 2022). Therefore, IMA/FEP might play a role as an organ-to-organ signal that moves from shoots to roots to promote Fe absorption (**Figure 1B**) (Grillet et al., 2018; Tabata et al., 2022). Although the mobility of IMA/FEP has not yet been cofirmed, it is possible that IMA/FEP mobilized from shoot tissues interacts with BTS/BTSLs within root vascular tissues, thereby stabilizing the IVc subgroup bHLH TFs. Since the bHLH POPEYE (PYE) has recently been shown to move between root cells (Muhammad et al., 2022), one possible model is that the IVc subgroup bHLH TFs moves from the vascular bundles to the epidermis to activate Fe uptake. However, further detailed analysis at the cellular and tissue level is needed, including whether IMA/FEP, a candidate systemic signal molecule, activates Fe absorption and coumarin biosynthesis *via* a mechanism similar to the local Fe-deficiency response.

Organ-to-organ signaling regulation is known to regulate root Fe absorption in response to the Fe-nutritional status of leaves, *i*.*e*., a directional shoot-to-root signaling regulation (Maas et al., 1988; Mendoza-Cózatl et al., 2014; Zhai et al., 2014). The concentration of Fe in the phloem regulated by OPT3 transporter plays a role in the shoot-root communication for Fe demand. In the Arabidopsis *opt3* mutant, all *IMA*/*FEP* genes were upregulated (García et al., 2022), suggesting that a shoot-derived systemic signal could act upstream of the *IMA*/*FEP* peptide signaling pathway. Fe concentrations in the phloem are also regulated by ammonium in a cell wall-localized ferroxidase LPR2-dependent manner (Liu et al., 2022). The regulation of systemic signaling *via* iron-nitrogen interactions is one of the interesting research areas that require further analysis. Arabidopsis possesses two systemic signaling pathways for N acquisition (Ota et al., 2020). The CEPD-like 2 (CEPDL2) pathway works from shoots to roots, depending on the shoot N status. When roots are subjected to severe N deficiency conditions, the CEPDL2 (shoot-to-root) and CEPD (root-to-shoot-to root) pathways are upregulated (Ota et al., 2020). Therefore, as with systemic N signaling, two pathways, the shoot-root or the root-shoot-root pathway, may be balanced by the shoot Fe status and rhizosphere Fe availability. Further verification of the relationship between these two organ-to-organ communication mechanisms is needed.

## Conclusion

IMA/FEP peptides have critical roles in Fe acquisition and homeostasis in angiosperms. Recent studies of IMA/FEP peptides have revealed that IMA/FEP peptides disturb the interaction between bHLH transcription factors and BTS/BTSLs, inhibiting the degradation of these transcription factors by BTS/BTSLs. This interaction is important for maintaining the Fe-deficiency response in roots. Our understanding of these peptide functions for systemic Fe signaling is incomplete. Precise spatiotemporal expression analysis of IMA/FEP peptides at the whole plant levels and further detailed biochemical analysis of peptides will provide novel insights into the molecular functions of IMA/FEP peptides in the Fe-acquisition system of plants.

## Author contributions

The author confirms being the sole contributor of this work and has approved it for publication.
